# Special Issue: Enzyme Immobilization 2016

**DOI:** 10.3390/molecules22040601

**Published:** 2017-04-08

**Authors:** Roberto Fernandez-Lafuente

**Affiliations:** Departamento de Biocatálisis, Instituto de Catálisis-CSIC, C/Marie Curie 2, Campus UAM-CSIC, Cantoblanco, 28049 Madrid, Spain; rfl@icp.csic.es; Tel.: +34-585-4941

The use of enzymes as industrial biocatalysts is currently a solution for many problems of modern organic chemistry, which tries to carry out the most complex reactions under the rules of green chemistry [1]. In this context, enzyme immobilization is a critical point for the implementation of many processes [2]. This technique has been developed to obtain a heterogeneous biocatalyst, and thereby to produce reusable enzymes. From this necessity, immobilization has evolved to solve some other limitations of enzymes, like stability, activity, selectivity or resistance to inhibitors [3], and even to improve enzyme purity [4].

However, only properly designed immobilization strategies, based on the understanding of the protein immobilization mechanisms, may be able to optimize these results: immobilization support, active groups in the support, immobilization protocol and enzyme-support reaction end point need to be carefully selected [2,3,4]. From this viewpoint, immobilization of enzymes, far from being an old-fashioned methodology to just reuse these expensive biocatalysts, has become a powerful tool to greatly improve the enzyme properties [2,3,4].

This interest justifies the interest raised by the Special Issues in “Enzyme immobilization” published in Molecules. To the 23 papers collected in the previous issue, this new issue gathers 20 new contributions, resuming some of the most significant advances in the field of immobilization of enzymes.

The reviews included in these Special Issues comprise very diverse topics. The possibilities of improving enzyme properties via immobilization, focusing on oxidoreductases, have been explained in a review paper [5] with a high impact in citations number. Other reviews have discussed the advantage of diverse materials for enzyme immobilization, like inorganic supports [6] or agarose beads [7]. Immobilization of enzymes in magnetic nanoparticles [8] has been also reviewed, showing how nanotechnology may open new opportunities to the immobilization of enzymes. In another review, the fusion of the enzymes of interest to polyhydroxyalkanoate with covalently attached synthase enzyme has been discussed as a method to achieve site-directed protein immobilization [9]. In a similar context, another review has focused on recent advances in enzyme engineering towards in situ self-assembly (bioengineering of bacteria to abundantly form enzymatically active inclusion bodies such as enzyme inclusions or enzyme-coated polyhydroxyalkanoate granules) [10]. Reviews also include the design of enzymatic biosensors for drug screening and pharmaceutical kinetic studies [11] and the immobilization of some glycoside hydrolases [12].

Some papers involve specific enzymes. For example, laccase has been immobilized in tailor-made siliceous ordered mesoporous materials [13], inulinase has been non-covalently immobilized on carbon nanotubes [14], organic/magnetic nanocarriers bearing hyperbranched poly(amido acids) were used to immobilize γ-glutamyltranspeptidase [15], wool activated by cyanuric chloride has been used to immobilize α-amylase [16], laccase has been immobilized on a pan/adsorbent composite nanofibrous membrane [17], a thermophilic esterase has been immobilized on an epoxy activated support [18], horseradish peroxidase has been attached to graphene oxide/Fe_3_O_4_ using 1-ethyl-3-(3-dimethyaminopropyl)carbodiimide as a cross-linking agent [19]. Comparison of differently immobilized enzymes has been the subject of some papers. For example, the performance of α-amylase from *Anoxybacillus* sp. SK3-4 immobilized on Relizyme or Immobead supports has been compared [20]. A neutral protease from *Bacillus subtilis* was immobilized on diverse support. Glyoxyl-agarose gave the best results and it proved to be a very useful biocatalyst to produce capecitabine [21]. Cellulase was immobilized via the CLEA technology using magnetic nanoparticles to facilitate its handling and used to hydrolyze lignocellulose [22]. Dehalogenase ST2570 has been site-specifically covalently immobilized utilizing a formylglycine-generating enzyme [23].

Lipase immobilization has been the focus of many contributions. Lipase from *Candida rugosa* has been immobilized in methacrylate-substituted polyphosphazene beads [24], lipase B from *Candida Antarctica* has been immobilized in hydrophobic core-shell supports [25], poly(ethylene glycol) decorated polystyrene nanoparticles modified by the adsorption of Congo red [26], styrene-divinylbenzene beads [27,28] or a collection of different hydrophobic supports [29] have been evaluated to immobilize different lipases. Prevention of enzyme desorption of lipases immobilized on hydrophobic supports have been subject of different papers. For example, glutaraldehyde-acyl heterofunctional mesoporous silica gel support has been used to immobilize the lipase from *Burkholderia cepacia* [30] or lipases from *Thermomyces lanuginosus* and *Pseudomonas fluorescens* [31], the immobilization proceeded first via lipase interfacial activation versus the acyl layer followed by the covalent attachment via glutaraldehyde. Heterofunctional octyl-amino agarose beads have been used to immobilize diverse lipases also aiming to prevent enzyme desorption while maintaining the advantages of the use of hydrophobic supports to immobilize lipases [32]. Finally, the coating of immobilized lipases with PEI have also been assayed to avoid enzyme desorption via physical crosslinking [33]. Later, the authors showed how unfolded enzyme/PEI composites were formed during thermal inactivations. This made the process of their desorption from the support more complicated when the authors intended to reuse a clean octyl-agarose support for a new immobilization [34]. Lipase B from *C. antarctica* has been also immobilized on bisepoxide-activated aminoalkyl resins showing the effect of the spacer arm on the performance of the final immobilized enzyme [35]. Co-immobilized lipase from *Candida rugosa* and magnetic nanoparticles using metal coordinated hydrogel nanofibers is also presented; the relative activity of the composite is 8-fold higher than the free enzyme under certain conditions [36].

In some instances, the emphasis of the papers has been focused on the improved application of the immobilized enzymes for a specific process. Special interest has been shown to the use of several immobilized enzymes to catalyze a cascade reaction. The authors have used individually immobilized enzymes to prevent the problems derived from the use of coimmobilized enzymes [37]. Thus, β-galactosidase from *Bacillus circulans*, l-arabinose (D-galactose) isomerase from *Enterococcus faecium* and d-xylose (d-glucose) isomerase from *Streptomyces rubiginosus* were immobilized individually onto Eupergit supports and employed to transform lactose into ketohexose [38].

In another paper, combi-CLEAs of β-glycosidases produced from the commercial preparation AR2000 (a mixture of diverse enzymes) was used to increase the amount of terpenes, linalool, nerol and geraniol in wine, and that way to improve its organoleptic properties [39].

Lipase from *Rhizopus oryzae* was covalently immobilized on sepiolite and employed in the production of a biofuel similar to biodiesel [40]. The degradation of 2,4-dichlorophenol was performed using horseradish peroxidase covalently immobilized via glutaraldehyde chemistry [41]. The multimeric structure of a nucleoside 2’-deoxyribosyltransferase was stabilized via immobilization onto different and chemical crosslinking, and the biocatalyst thus prepared utilized in the synthesis of nucleoside 2'-deoxyadenosine from 2'-deoxyuridine and adenine [42]. The thermophilic esterase from *Archaeoglobus fulgidus* was adsorbed on hydrophobic Sepabeads EC-OD and the immobilized enzyme was further incubated in the presence of glutaraldehyde and successfully employed in the synthesis of poly(ε-caprolactone) [43]. Lecitase was immobilized on styrene-divinylbenzene beads and utilized under ultrasound stirring as catalyst in the synthesis of flavor esters [27]. Changes in enzyme specify upon immobilization are discussed in several papers [28,29,31,32,44].

In a very elegant approach, coimmobilization of lysozyme with a target enzyme (in the example a β-galactosidase) was shown to prevent enzyme degradation by bacterial attack [45]. In this example, enzyme coimmobilization is compulsory, even with the problems that this may generate [37].

The development of techniques to visualize the immobilized enzymes may provide interesting information to understand these processes and may open new opportunities to improve the control and understanding of the enzyme immobilization following different protocols. In this sense, confocal microscopy was used to identify the distribution of enzymes trapped in alginate [46]. In another paper, immobilized enzymes were used to develop implantable glutamate sensors [47].

Finally, a commentary paper was dedicated to a protein engineering approach as an alternative to immobilization, using a recombinant silk protein into which metal active sites can be incorporated to produce solid-state metalloprotein materials [48].

I hope that the collection of papers gathered in these two issues provides a vision of the current and future trends in the development of enzyme immobilization and biocatalysis. These special issues aim to point to the great potential of enzyme immobilization to solve some enzyme limitations and encourage future research in this area, and hopefully this second issue will not be the last one on this matter.
